# Ten simple rules to ruin a collaborative environment

**DOI:** 10.1371/journal.pcbi.1009957

**Published:** 2022-04-14

**Authors:** Carolyn J. Lawrence-Dill, Robyn L. Allscheid, Albert Boaitey, Todd Bauman, Edward S. Buckler, Jennifer L. Clarke, Christopher Cullis, Jack Dekkers, Cassandra J. Dorius, Shawn F. Dorius, David Ertl, Matthew Homann, Guiping Hu, Mary Losch, Eric Lyons, Brenda Murdoch, Zahra-Katy Navabi, Somashekhar Punnuri, Fahad Rafiq, James M. Reecy, Patrick S. Schnable, Nicole M. Scott, Moira Sheehan, Xavier Sirault, Margaret Staton, Christopher K. Tuggle, Alison Van Eenennaam, Rachael Voas

**Affiliations:** 1 Department of Agronomy, Iowa State University, Ames, Iowa, United States of America; 2 Department of Genetics, Development and Cell Biology, Iowa State University, Ames, Iowa, United States of America; 3 Research, National Corn Growers Association, Chesterfield, Missouri, United States of America; 4 Department of Agricultural Economics, University of Wisconsin, River Falls, Wisconsin, United States of America; 5 Filament, St. Louis, Missouri, United States of America; 6 Plant Soil and Nutrition Research Unit, USDA Agricultural Research Service, Ithaca, New York, United States of America; 7 Department of Statistics, University of Nebraska, Lincoln, Nebraska, United States of America; 8 Department of Food Science and Technology, University of Nebraska, Lincoln, Nebraska, United States of America; 9 Department of Biology, Case Western Reserve University, Cleveland, Ohio, United States of America; 10 Department of Animal Science, Iowa State University, Ames, Iowa, United States of America; 11 Department of Human Development and Family Studies, Iowa State University, Ames, Iowa, United States of America; 12 Department of Sociology and Criminal Justice, Iowa State University, Ames, Iowa, United States of America; 13 Research and Business Development, Iowa Corn Promotion Board, Johnston, Iowa, United States of America; 14 Department of Industrial and Manufacturing Systems Engineering, Iowa State University, Ames, Iowa, United States of America; 15 Department of Psychology, University of Northern Iowa, Cedar Falls, Iowa, United States of America; 16 School of Plant Sciences, University of Arizona, Tucson, Arizona, United States of America; 17 Department of Animal, Veterinary and Food Sciences, University of Idaho, Moscow, Idaho, United States of America; 18 Research and Development, Nuseed Canada, Laverton, Canada; 19 Agricultural Research Station, Fort Valley State University, Fort Falley, Georgia, United States of America; 20 Department of Animal Science, University of Florida, Gainesville, Florida, United States of America; 21 Institute of Biotechnology, Cornell University, Ithaca, New York, United States of America; 22 Agriculture and Food, CSIRO, Canberra, Australia; 23 Institute of Agriculture, University of Tennessee, Knoxville, Tennessee, United States of America; 24 Department of Animal Science, University of California-Davis, Davis, California, United States of America; Dassault Systemes BIOVIA, UNITED STATES

## Introduction

Trigger warning: Here, you will find a bit of satire, written from the not-so-funny, real experiences of the authors who have been involved in “team science” collaboratives. The material presented below covers topics that readers may find offensive or even traumatizing. We present a breakdown (pun intended) of how to ruin a functioning collaboration, rather than how to build one.

The ideas contained in this work were developed during two virtual meetings of members of the Agricultural Genome to Phenome Initiative (AG2PI; www.ag2pi.org) community and leadership team in May and June of 2021. In these sessions, we looked back at collaborative projects that were miserable failures and recalled what went wrong so we could avoid making the same mistakes in the future. We also considered what signals we might have missed that could have saved us some misery and where we might have had some blind spots (but should have seen coming).

As a side note, having worked on dysfunctional teams from time to time, we found writing this set of rules to be both cathartic and vastly cheaper than therapy. If you are not prepared for what will likely be the occasional, “Yikes, that sounds terribly familiar!” or would rather read some more upbeat advice, here are a few options we recommend: Vicens and Bourne [1], de Grijs [2], Knapp and colleagues [3], Cechova [4], Sahneh and colleagues [5], and Gewin [6].

### Ten simple rules

#### 1. State lofty goals, provide inadequate resources

**Fig 1:**
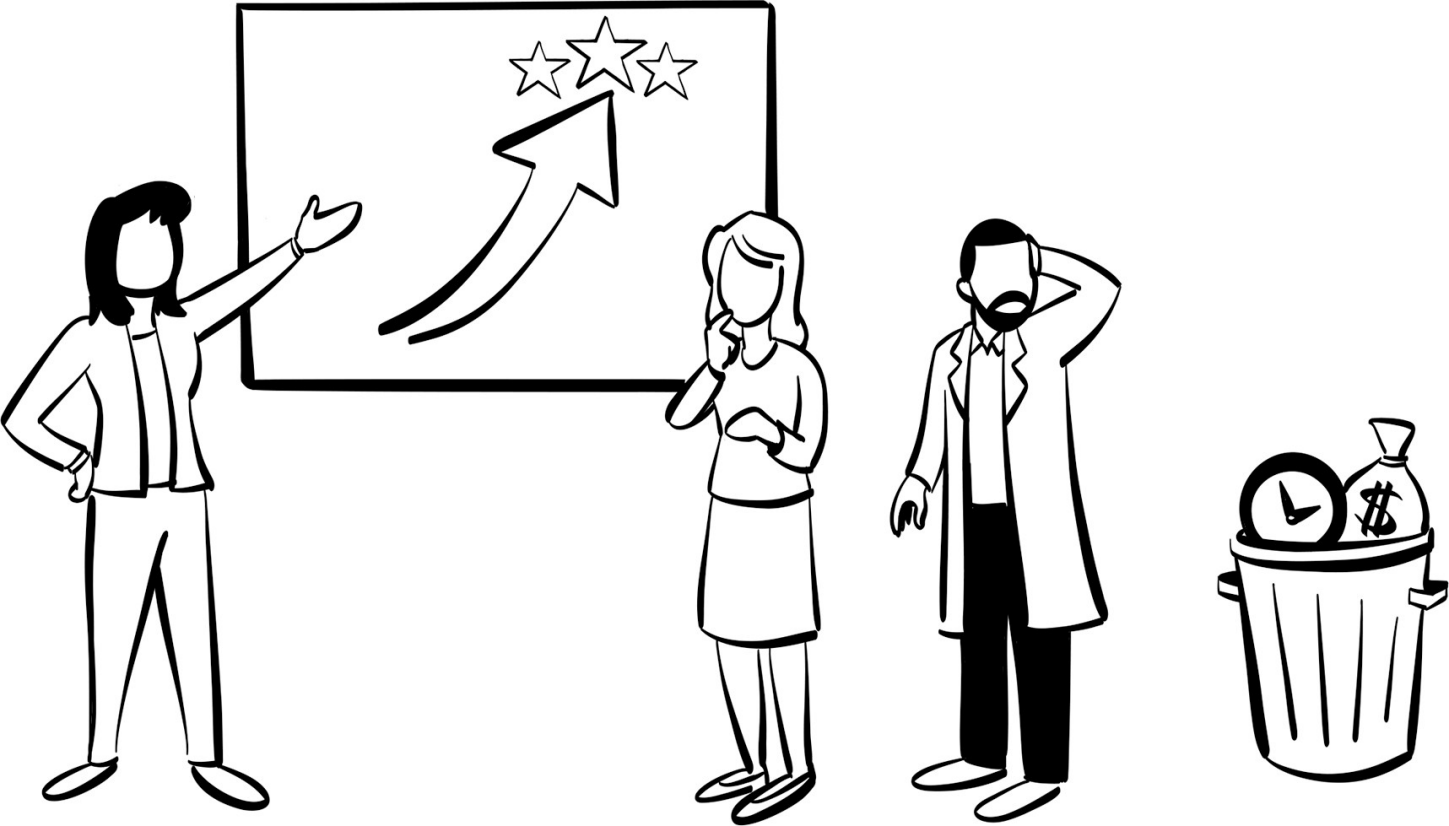
No need for time and money—we’re EXCITED!

How can you set a team up for disaster from the very beginning? Start with a big vision such as “Genome-in-a-spreadsheet promised in 5 months!” or “Will end world hunger in 90 days!” then provide few resources, ensuring the available resources are neither adequate nor appropriate. Some teams are energized by the promise of solving a big problem so ensure the team cannot do their best job—eliminate any available time and funding specific to the cause. By removing at least one of those resources, only a second-rate job is possible, at best. With an insufficiency of both time and funding, a third-rate job is the most likely result. Add to the mix insufficient or poorly matched knowledge, skills, and abilities to accomplish stated goals, and the team will be hamstrung in no time. (Fig 1)

#### 2. Fail to consider that team members have competing priorities and demands on their time

**Fig 2:**
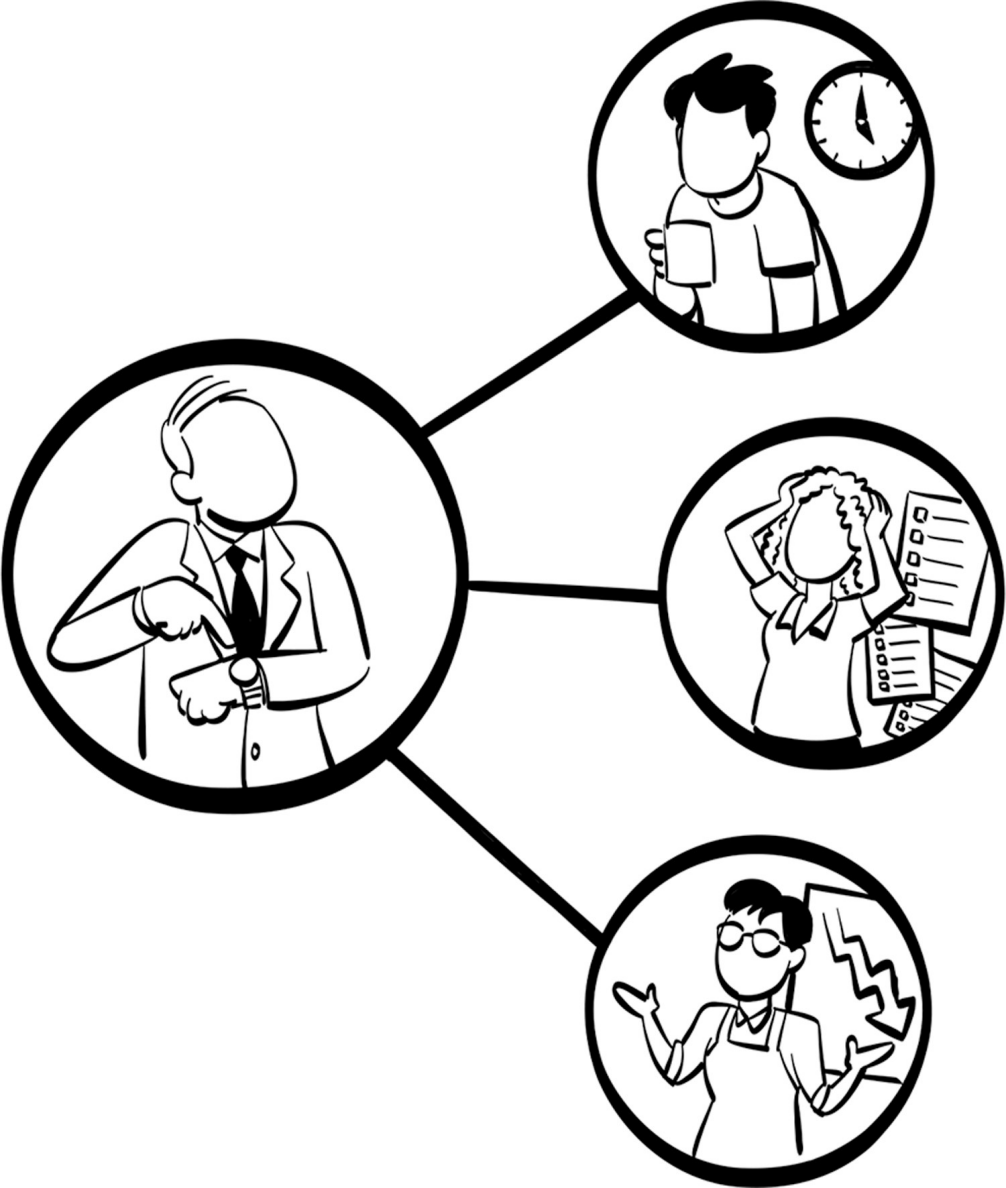
Personal boundaries? Disloyal.

Organize meetings that span weekends or require out-of-state travel and fail to consider the different time zones of team members. Nine AM Eastern time suits several of the senior team members, so this would be the ideal start for a meeting. Those unable to attend at 6 AM on the West Coast or 10 PM in Japan clearly lack commitment and setting up a way for members to attend virtually is troublesome. If someone is behind on their deliverables as a result of personal leave, they also clearly lack commitment. Further stratify team members by placing the utmost importance on the “hard” or “natural” science aspects of the project since this is obviously what the funding is REALLY meant to support. Schedule the social, or “soft” sciences and public impact, communications, and outreach discussions for late in the day; the real science topics will have been fully vetted by then so the real scientists can catch that early flight home: leave the soft stuff for the “others.” Prioritize publishing in a high-impact journal that focuses on science and technology; those working on social, behavioral, and humanities aspects can publish their work later, perhaps at the same time that the outreach team translates the project data into something useful. (Note: This assumes that any funding actually remains for these aspects of the project once the real science is done.) (Fig 2)

#### 3. Encourage dysfunction and animosity to develop and persist

Teams are inherently social, which means they are susceptible to dysfunctional social organization (so this step should be quite easy!). Unsuccessful teams fail to recognize that connection and collaboration are the lifeblood of teams working to solve complex problems. Want to encourage dysfunction in your research team? Take your finger off the pulse of the team and instead fly blind to the mood among your members. Enable disciplinary and methodological factions to take root and flourish. Fail to plan for interpersonal conflict and cantankerous individuals within your teams. Should a team member provide allegations of sexual misconduct or harassment, do not take action or inform the proper authorities. Devote all your team time and resources to the technical aspects of the grant/contract, rather than that interpersonal fluff. Everybody hates icebreakers and other team building exercises anyway, so you are doing the team a favor.

Beware: Not all types of conflict will sink your team! There is such a thing as productive conflict, so keep your wits about you as you nurture dissent. Diverse teams often perform better because they feel *less* comfortable with one another than homogenous teams [7]. In these diverse settings, individuals must work harder to develop solutions that convince a broader set of stakeholders. As a result, they often identify more novel approaches, see greater breakthroughs, and provide a competitive edge over other teams.

**Fig 3:**
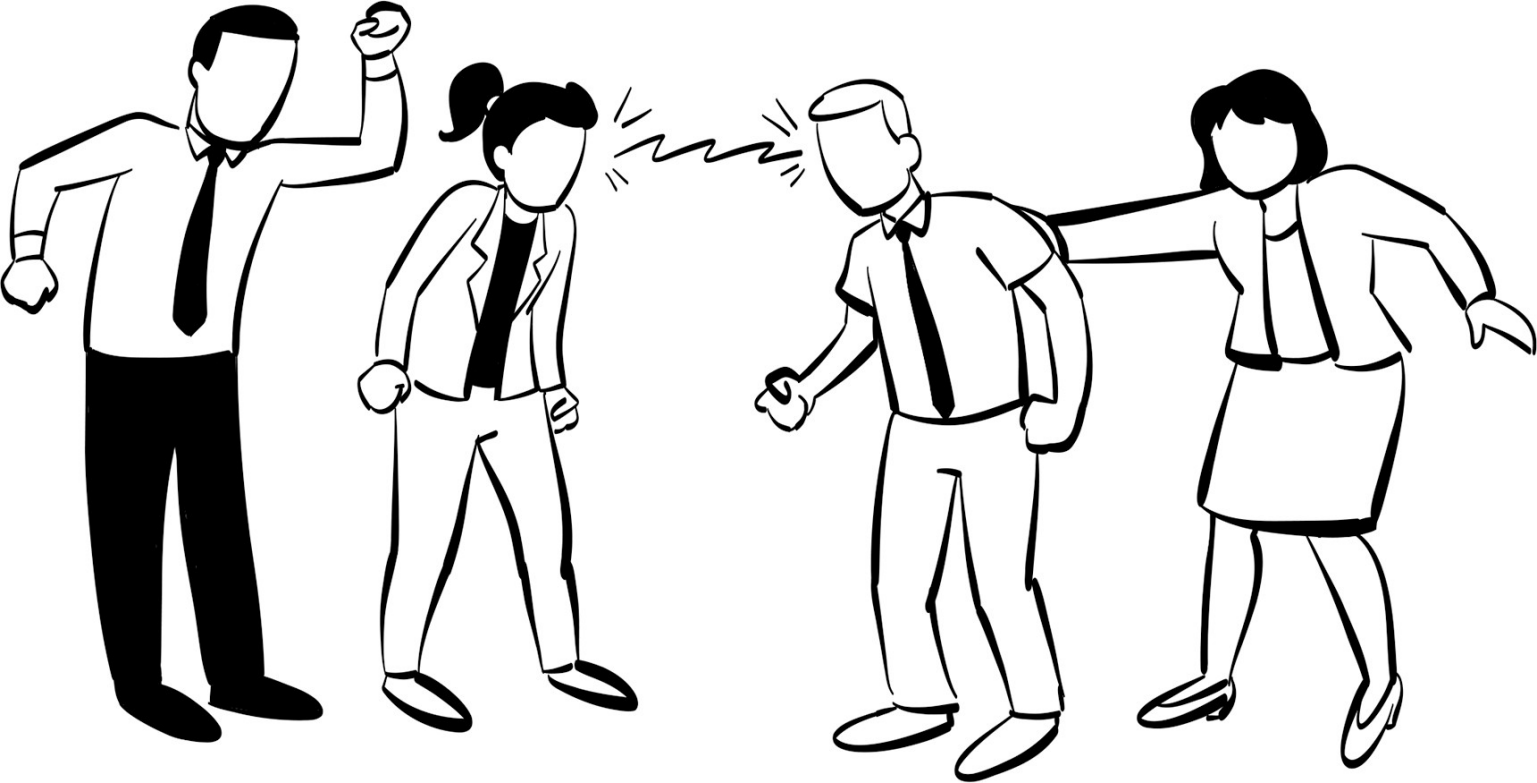
Fight! Fight! Fight!

To harpoon a diverse team that manages conflict well, focus on differences in values rather than ideas, never ask people to “see it from another perspective,” and, whatever you do, don’t create an atmosphere where people feel welcomed and respected. By encouraging disagreement (or simply by allowing it to fester), you can create an environment of dysfunction, conflict, and division. (Fig 3)

#### 4. Plan lots of meetings and normalize poor attendance. Fail to act on meeting outcomes, citing low attendance

**Fig 4:**
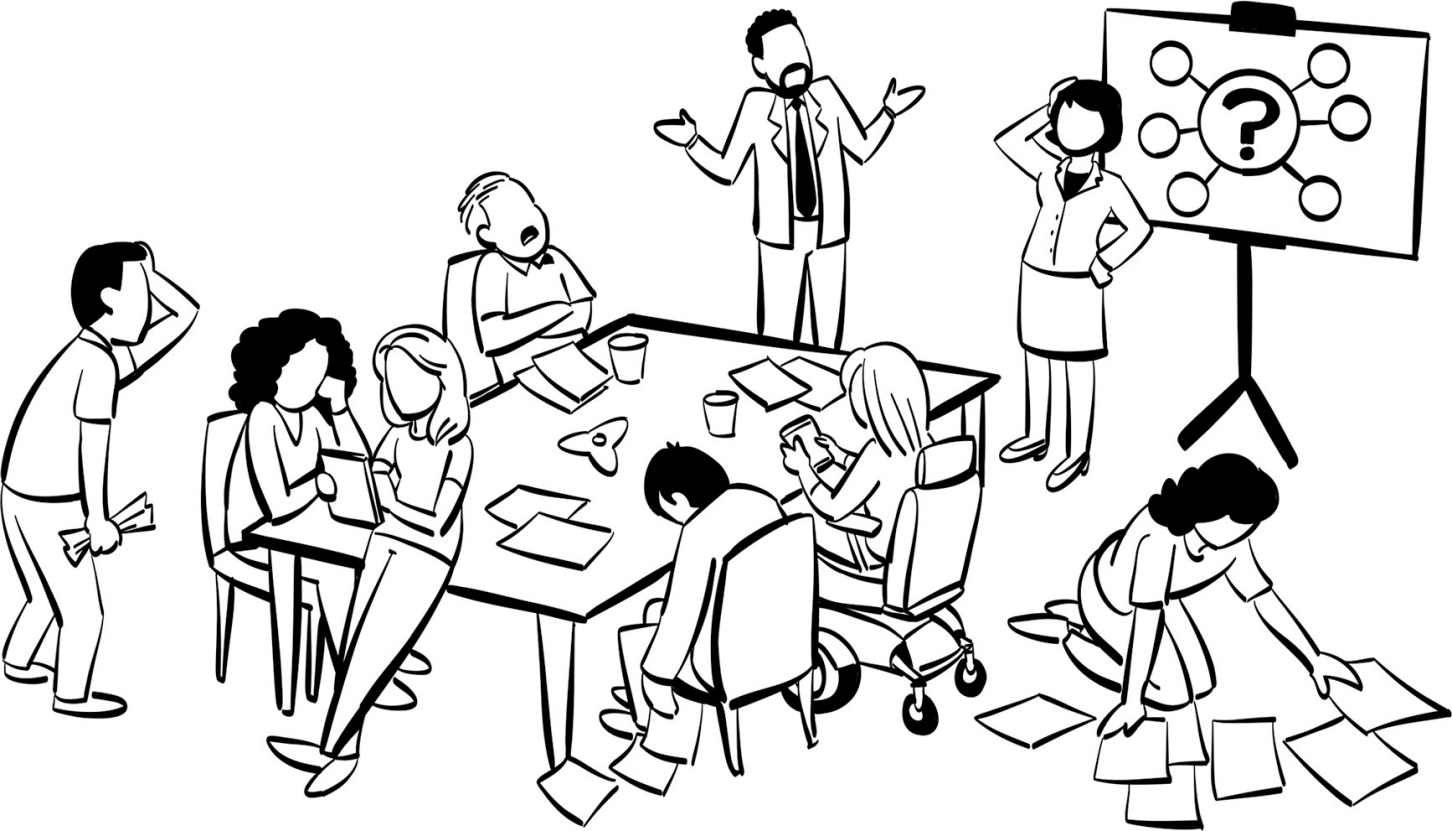
Death by meeting.

Schedule frequent group meetings that are neither prioritized nor well attended. Make sure that these meetings are scheduled for an hour but make sure they run long each time. Before each meeting, there should be no appointed person to lead the meeting, no agenda, and any items discussed should be talked about in vague terms without defined priorities or next steps. Gregarious individuals should be encouraged to speak freely and to frequently interrupt other individuals, especially if a valid point or concern is about to be raised. For the latter case, make sure the individual makes a dismissive comment during their interruption to ensure the person does not try to continue to speak. Do not tolerate inclusive meetings and make sure people who are normally quiet remain quiet and are never given the opportunity to present their views, opinions, or ideas. As team members stop attending these meetings, make a rollcall, and make obvious notes of people who are not present. When problems develop that are too large to ignore, hold large meetings to discuss and identify an arbitrary set of people to hold responsible for the failure. Make sure not to inform these people prior to the meeting to ensure inability to prepare a thoughtful defense (e.g., “that isn’t my expertise”) or, even worse, actually solve the problem. If the meeting to identify a scapegoat starts to resolve issues, emergency procedures could involve, for example, yelling out about something random; referring back to your graduate school experience; or sharing unrelated stories to run out the clock on the meeting before anything gets truly resolved. (Fig 4)

#### 5. Misalign expertise, skills, and abilities of group members with specific and unrelated goals and deliverables

**Fig 5:**
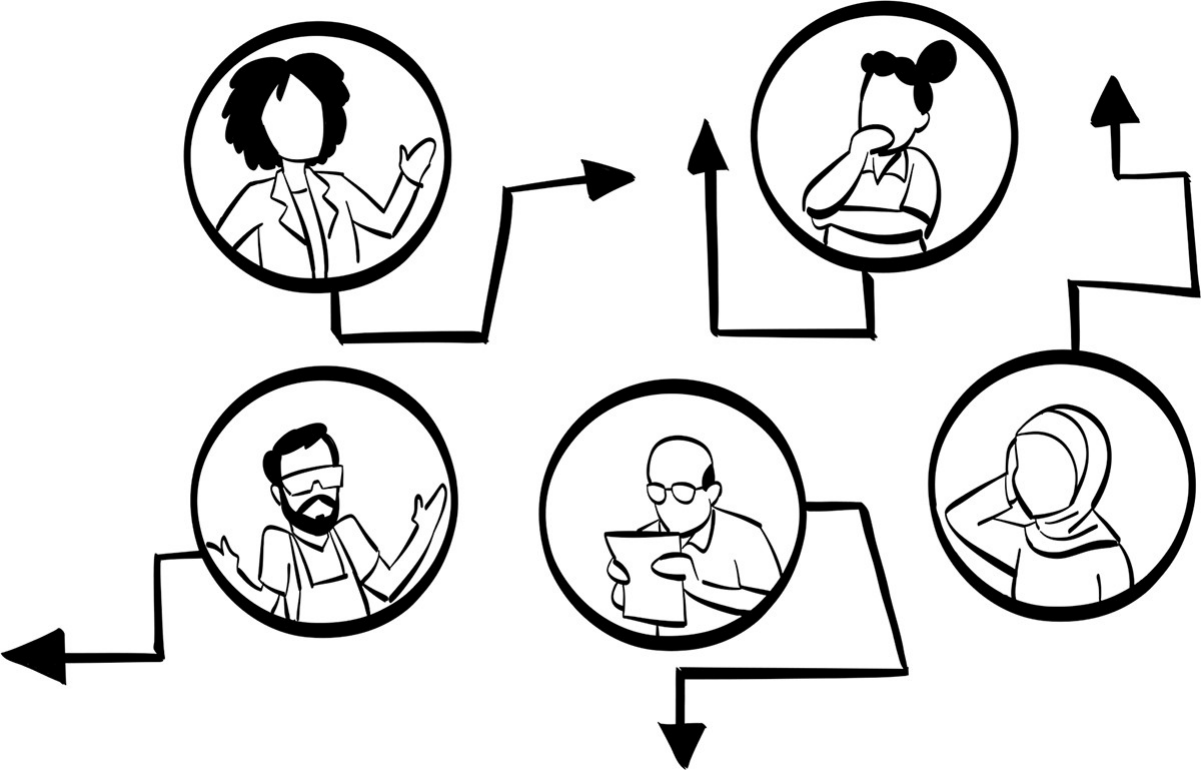
Who does what around here?

Building a team of experts is not the same as building an expert team. An expert team has alignment between a person’s skills and their deliverables. Intentional alignment is a management technique called putting “aces in their places.” The meaning is clear to anyone familiar with card games—aces can be useful wildcards, but only where their strengths improve the value of your hand for a particular game. Be sure to put those precious wildcards into the wrong situations so that their value is not fully achieved. To do this well, bring in great experts and aim their efforts at topics that don’t align with their skills and abilities. The result? Wild frustration for those wildcards! Team heterogeneity extends beyond just scientific training, functional skills, and interpersonal abilities. When hidden talents are revealed, fail to leverage those abilities toward the larger team goals. Your failure to reorganize teams and readjust team strategy is a great recipe for underperformance. (Fig 5)

#### 6. Fail to make changes to project plans over time. You had a specific plan initially, so stick with it no matter what!

**Fig 6:**
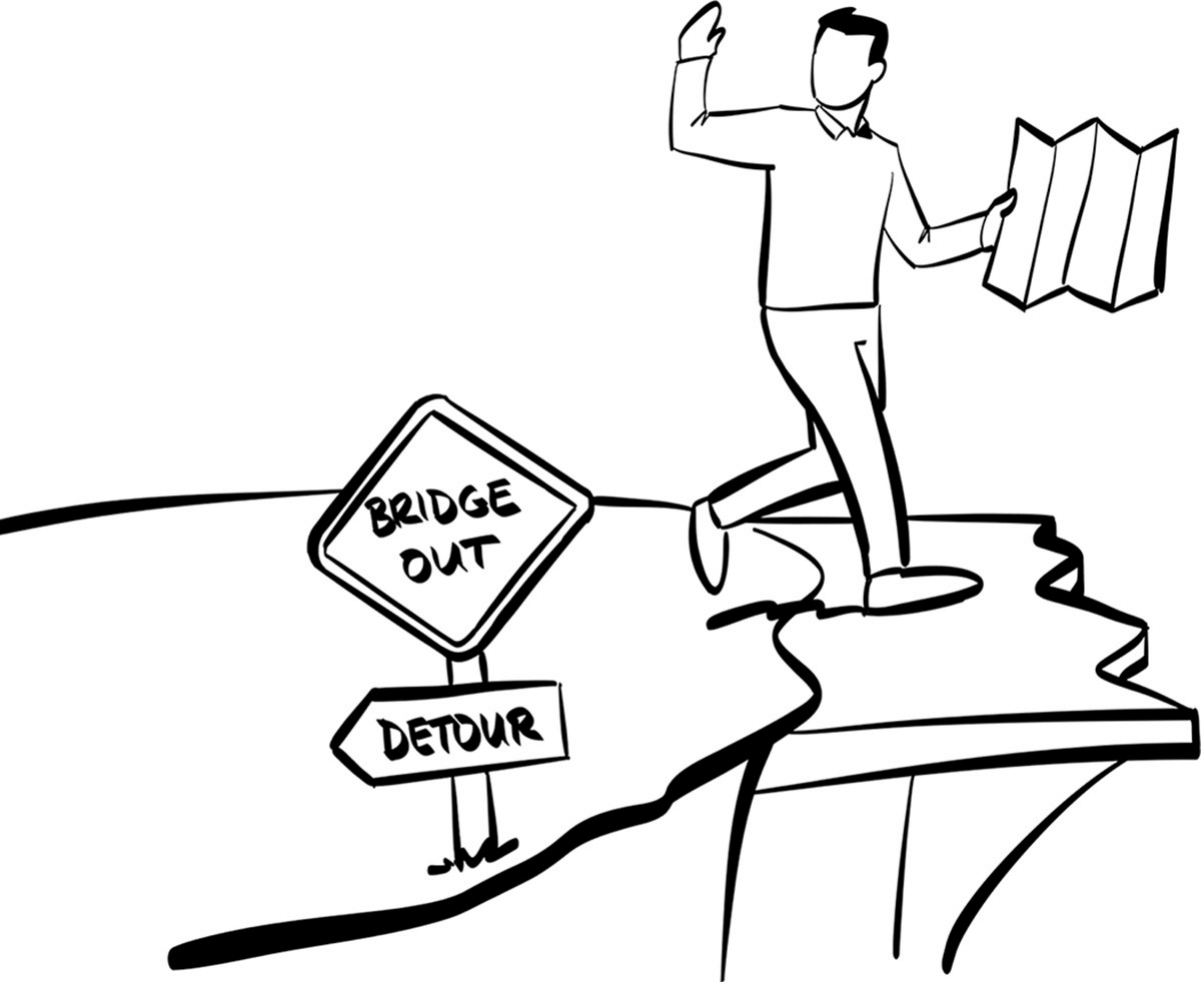
Never rethink the plan.

Over the life of a 3-to-5-year project, your vision and mission may not change dramatically, but knowledge and technologies will. This is bothersome because it means that some may begin to think your perfectly planned strategies and tactics have become outdated. Consider that sort of assessment to be a statement of disloyalty and say so! Elevate those who will execute on the original plan to positions of leadership and reward them for their team spirit. If you find that you can’t put a stop to progressive ideas entirely, slow things down. Changes won’t be useful if they are agreed upon and communicated too late in the game. This tactic is particularly useful because it enables you to point out that the idea to institute changes was clearly misguided given that the project ended up failing anyway! It’s a win-win. If all else fails and the team does succeed in enacting changes to project tactics, there is a failsafe: Don’t inform your project manager because they will almost certainly try to help roll out the changes, which would derail the confusion and dysfunction you seek to create. (Fig 6)

#### 7. Include in the leadership team giants in the field who have no bandwidth to execute on deliverables

**Fig 7:**
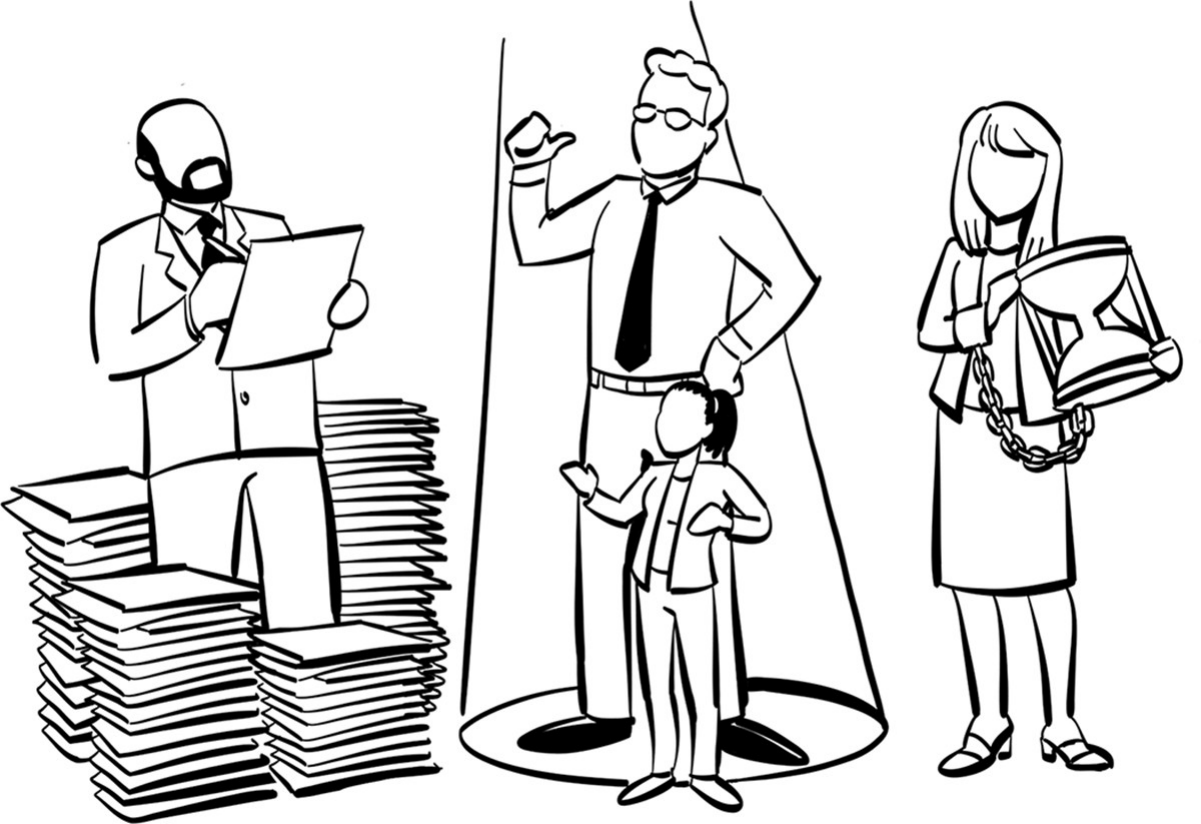
Limelight on the bigshot.

Thought leaders notoriously show up to a collaboration excited, then become scarce when there’s work to be done. When considering Lencioni’s “Five Dysfunctions of a Team,” these special snowflakes can be counted on to deliver a twofer: they lack commitment ***and*** fail to deliver results [8]. As an added bonus, they model these behaviors to other budding sociopaths on the team. The key to finding these individuals? Pay attention to those who keep reminding others of how busy they are, even though they seem to find time to contribute to prestigious opportunities that arise. To enable maximal impact of this individual for crushing team spirit, avoid including strong, communicative team players with project management skills. Those who think they can define clear, achievable deliverables are just a pain in the neck and will irritate your big-name team members to the point of abandoning the project for more high-profile projects. (Fig 7)

#### 8. Make sure the team’s leader is an insecure micromanager who believes rules only apply to those lower in the power dynamic

**Fig 8:**
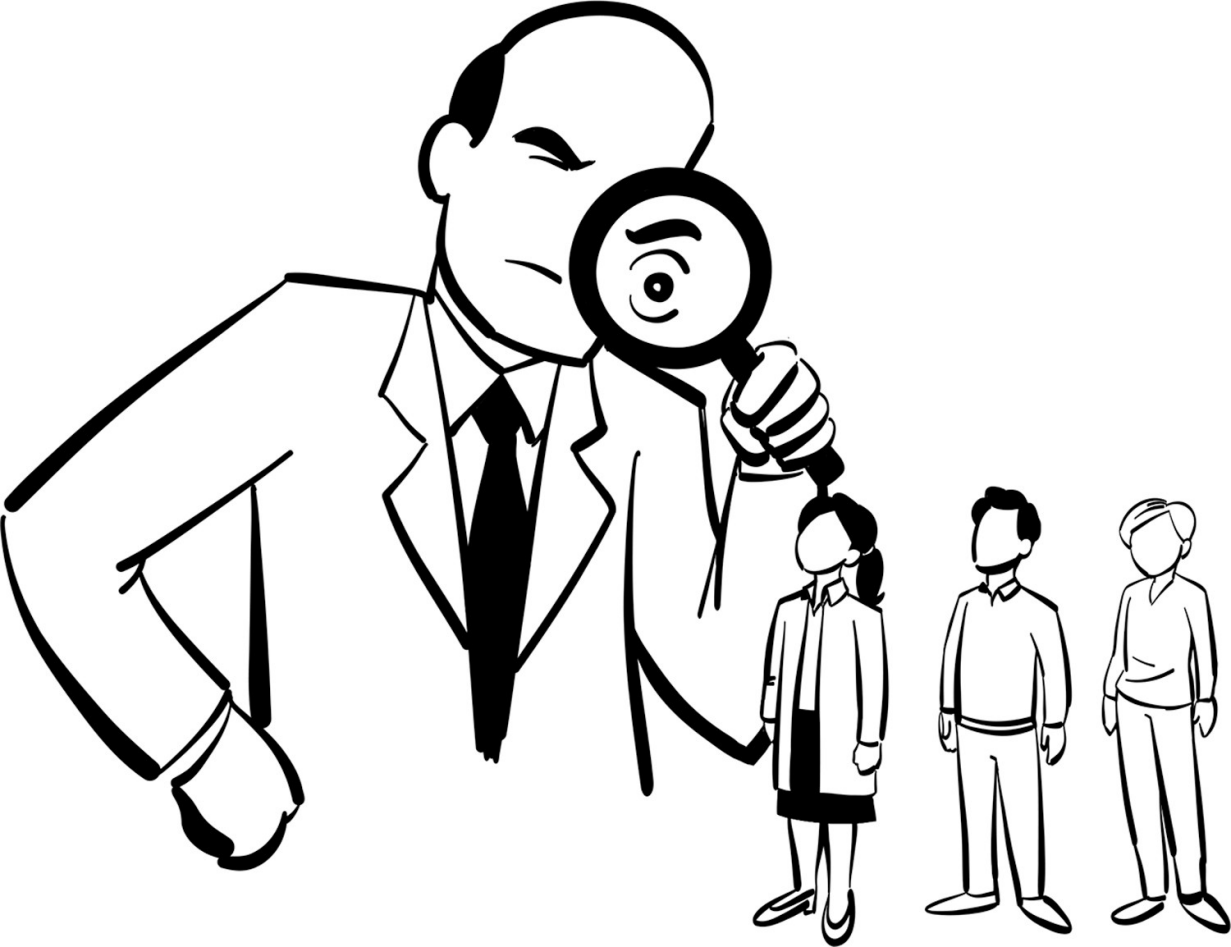
Micromanagers can have a soul-crushing impact!

Bringing together a team of experts and not allowing them to contribute their voices, experiences, and knowledge, in particular team members who are of lower status, is a sure way to ruin a collaborative environment. When collaborators are micromanaged and not permitted or empowered to lead in their respective areas, the project will often fail to reach its full potential. Find a team manager who has insecurities, irrational fears, and a fragile ego, so they can lead the team down a path of “my way or the highway.” These types of micromanagers lead from the misconception that there is only one way to be successful: their way. Because the micromanager will almost certainly find that the rest of the team is incompetent, they will establish new rules and guidelines “for the greater good of the project” but of course, only expect the rest of the team to follow them. A successful micromanager will also make all budgetary decisions in secret, refusing to ask team members for input on how to prioritize spending decisions, because they know that other members are too busy (and too ignorant) to weigh in on such mundane matters. “The One Right Path” viewpoint is especially helpful when it squashes diverse opinions and reinforces predominant stereotypes for leadership in your particular region, research field, etc. (e.g., CIS males). Depriving collaborators of trust and respect while not acknowledging the value of their experiences is a sure way to ruin a good and mutually beneficial collaboration. (Fig 8)

#### 9. Treat individuals with unique expertise like they are different and/or don’t belong

**Fig 9:**
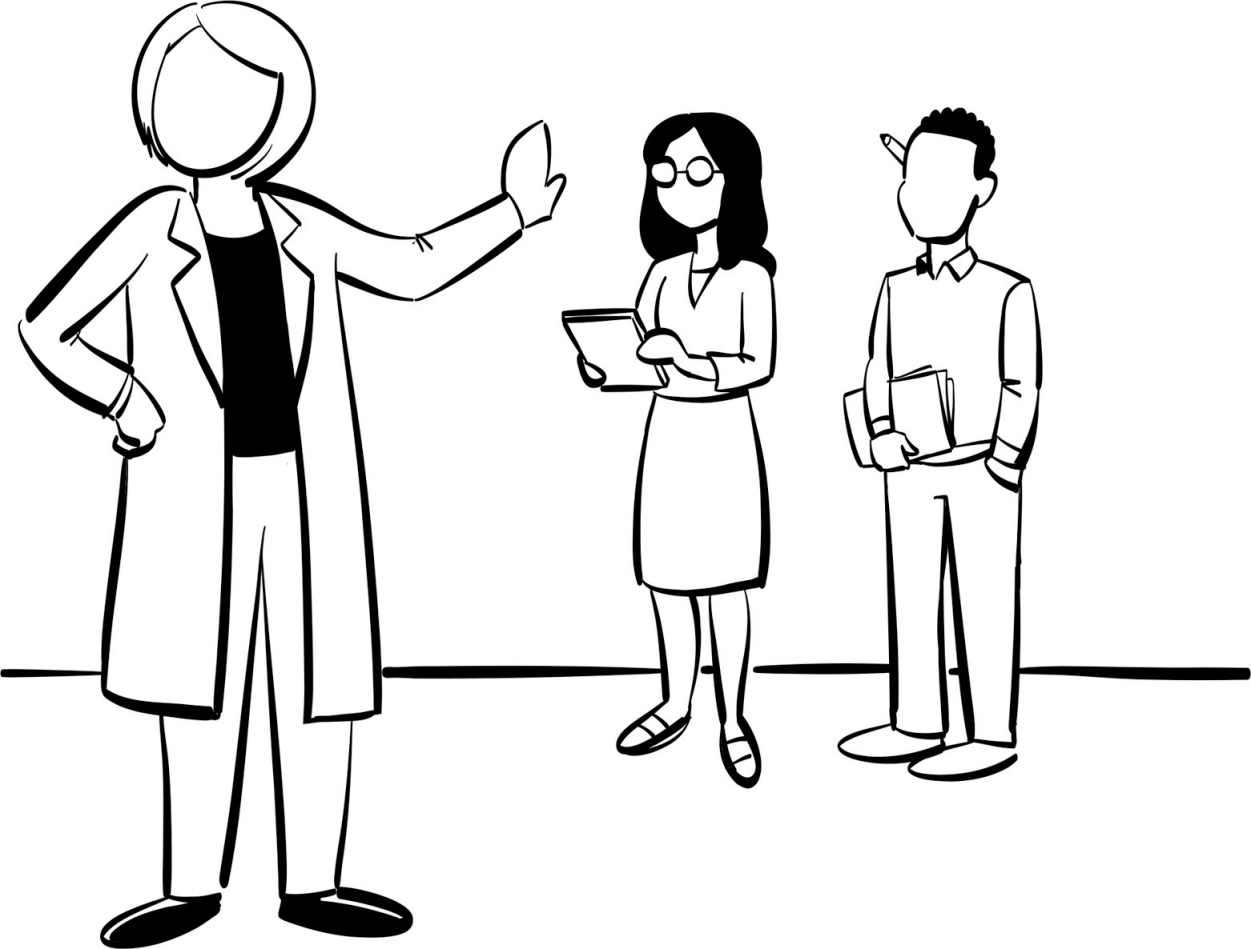
Who are these weirdos and why are they talking?

The newest fad in science is creating interdisciplinary (even *trans*-disciplinary) teams, with the aim to achieve research “consilience.” Those who support this sort of team building create research teams that are composed of individuals with unique yet complementary expertise and build a context and team dynamic in which they can work together successfully by standing on equal footing for engaged discourse and achievement. How can you use this knowledge to ruin a team? Don’t do it! Instead, follow Dr. Seuss’ lead and only give star status to a select few [9]. Who deserves star status? Team members who are the most cutthroat and those who are well connected. Don’t allow star status to be assigned to the best leaders, the most deserving, or members who are on the team merely to provide a “service.” The service workers—you know, data scientists, social scientists, statisticians, outreach professionals, and other staff—should not be treated as equal partners. Why? Because this approach will create imbalances in the team. Star members will become viewed as “core” and non-stars as “accessory.” It will become clear to all that core members deserve to be there so their contributions are valuable and essential; the accessory members should be made to feel that it’s a privilege to be included, but that they are to be seen, not heard. Eventually, accessory members should become disenchanted with the group’s big goals and will find excuses to disengage. Their potential contributions and expertise will be lost, and the potential for translation of the research results and impact will dwindle rapidly. (Fig 9)

#### 10. Fail to hear, consider, and respect ideas and dissenting opinions—especially from those who have diverse expertise and lived experience or who are early in their careers

**Fig 10:**
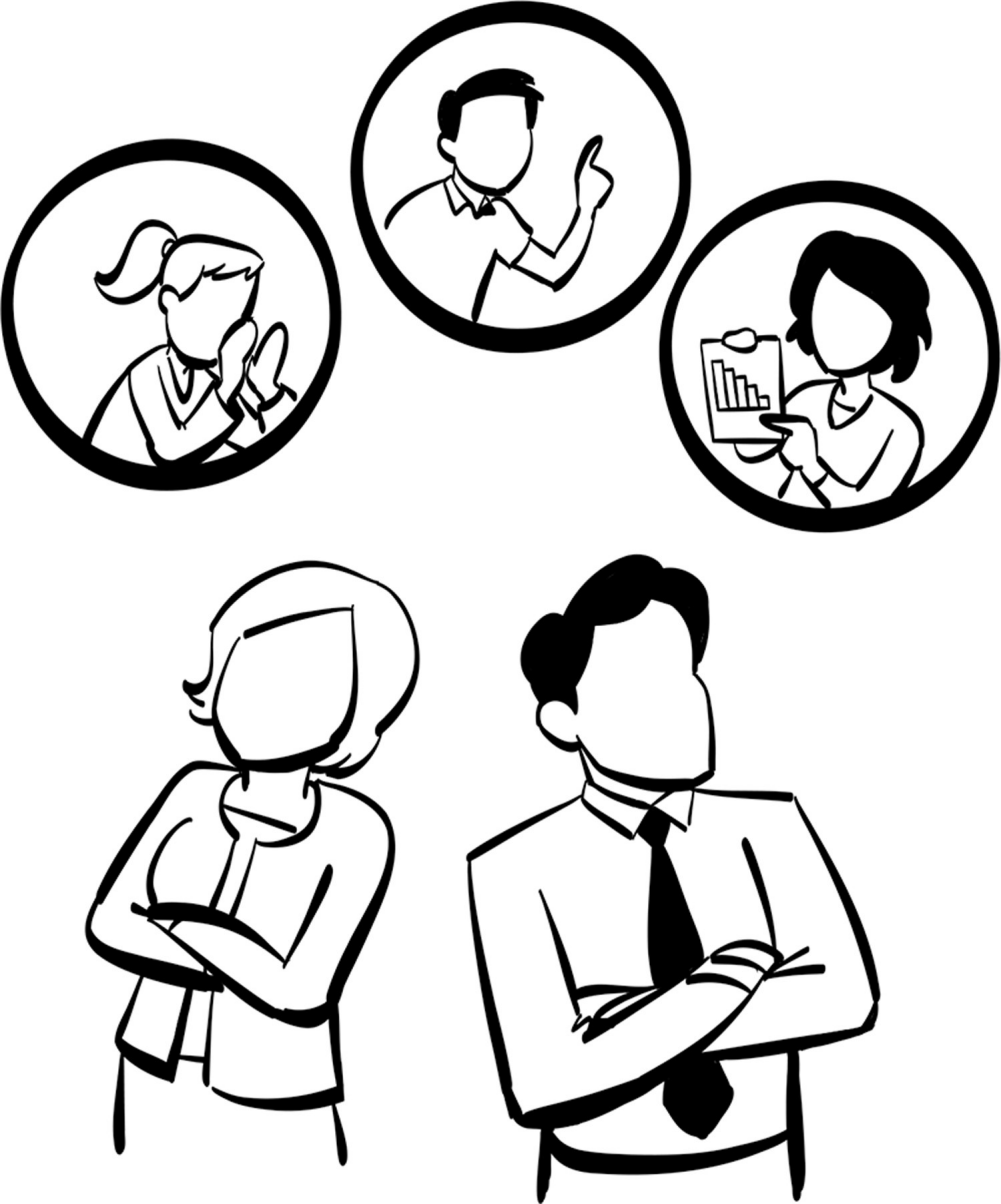
Just ignore them and maybe they’ll go away…

Make group members feel self-conscious or unworthy based on how their identities differ from the leader or, perhaps, from others in the group. You could comment on how your expectations differ for group members based on their racial [10], gender [11], or parental [12] identities. You could also try adding casual sexist or racist jokes into your repertoire; if that doesn’t do the trick, then you can point out team members “extra” time off for religious holidays or parental leave. Be sure to schedule all-hands meetings during these times to drive the joke home. Face it: You only included those people on the team because the request for proposals required it, anyway. Don’t listen to them. Dismiss their input! *You* are the expert. Yes, these team members were included expressly to provide valuable input, but we all know they were only included to check a box. We “know” our space! We’ve been (wildly) successful and what in the heck would someone with far less experience in this specialized area be able to add anyway? Of course, the *idea* was that these new team members from other disciplines and experiences would add value precisely because they have “fresh eyes” and new perspectives, but weren’t we idealistic once, too? Those people and their ideas require time and energy to discuss, vet, and incorporate into the project. Not worth it! It’s best to just list them on the proposal and then allow them to observe and “learn,” rather than engage. Not only will that tactic keep them from wrecking our paradigms, it will put them in their place—which is really somewhere else. Not here. Goodbye! (Fig 10).

We hope that by following these 10 simple rules, you will feel more confident to efficiently ruin collaborative projects. In fact, these rules are not just for individuals but for funding bodies as well, particularly with respect to writing the policies and regulations that guide the development of proposals and their assessment.

Although these 10 behaviors are the ones that we, the authors, agree are the most effective, based on our misfortunes, there are many other ways to ruin a collaboration! For science (and society), the opportunities to wreck people and their good science are nearly endless.
